# *Plasmodium falciparum* Merozoite Invasion Is Inhibited by Antibodies that Target the PfRh2a and b Binding Domains

**DOI:** 10.1371/journal.ppat.1002075

**Published:** 2011-06-16

**Authors:** Tony Triglia, Lin Chen, Sash Lopaticki, Chaitali Dekiwadia, David T. Riglar, Anthony N. Hodder, Stuart A. Ralph, Jake Baum, Alan F. Cowman

**Affiliations:** 1 The Walter and Eliza Hall Institute of Medical Research, Melbourne, Australia; 2 Department of Biochemistry and Molecular Biology, Bio21 Molecular Science and Biotechnology Institute, University of Melbourne, Australia; 3 The Department of Medical Biology, University of Melbourne, Melbourne, Australia; Case Western Reserve University, United States of America

## Abstract

*Plasmodium falciparum*, the causative agent of the most severe form of malaria in humans invades erythrocytes using multiple ligand-receptor interactions. The *P. falciparum* reticulocyte binding-like homologue proteins (PfRh or PfRBL) are important for entry of the invasive merozoite form of the parasite into red blood cells. We have analysed two members of this protein family, PfRh2a and PfRh2b, and show they undergo a complex series of proteolytic cleavage events before and during merozoite invasion. We show that PfRh2a undergoes a cleavage event in the transmembrane region during invasion consistent with activity of the membrane associated PfROM4 protease that would result in release of the ectodomain into the supernatant. We also show that PfRh2a and PfRh2b bind to red blood cells and have defined the erythrocyte-binding domain to a 15 kDa region at the N-terminus of each protein. Antibodies to this receptor-binding region block merozoite invasion demonstrating the important function of this domain. This region of PfRh2a and PfRh2b has potential in a combination vaccine with other erythrocyte binding ligands for induction of antibodies that would block a broad range of invasion pathways for *P. falciparum* into human erythrocytes.

## Author Summary

The causative agent of the most severe form of malaria in humans is the protozoan parasite *Plasmodium falciparum*. These parasites are carried by a mosquito that infects humans during feeding resulting in injection of sporozoite forms that infect and develop in the liver into the merozoite stage. The merozoites are released into the blood stream where they invade erythrocytes in which they can grow and divide. Invasion of the red blood cell by *P. falciparum* merozoites involves a cascade of protein-protein interactions. The *P. falciparum* reticulocyte binding-like homologue proteins (PfRh or PfRBL) are an important protein family involved in binding to specific receptors on the red blood cell. We have analysed two members of this protein family, PfRh2a and PfRh2b, and show that they undergo a complex series of cleavage events before and during merozoite invasion. We have defined the region of these ligands that bind red blood cells and show that antibodies to this receptor-binding region block merozoite invasion demonstrating the important function of this domain.

## Introduction

Invasion of apicomplexan parasites into host cells is a complex process involving multiple ligands stored in apical organelles known as micronemes and rhoptries (for review see [1]). The ligands are released from these compartments onto the invasive zoite form of the parasite during egress or invasion of the host cell where they are able to bind receptors. After initial contact involving low affinity interactions the parasite reorients so that the apical end is abutting the host cell membrane and a tight junction is formed with the invading parasite membrane. The tight junction involves specific parasite ligands and this structure is ultimately linked to the actomyosin motor that provides the force required for invasion (see for review [2]). Entry into the host cell is mediated by movement of the tight junction across the surface to the posterior, where membrane fusion completes formation of a parasitophorous vacuole surrounding the internalised parasite.

Whilst some apicomplexan parasites, such as *Toxoplasma gondii*, are able to invade many different host cells *Plasmodium spp.* merozoites have an exquisite preference for red blood cells and this is mediated by specific parasite ligand-host receptor interactions. In the case of *P. falciparum*, the causative agent of the most severe form of malaria in humans, this involves at least two protein families. Firstly, the erythrocyte binding-like (EBL) proteins have been shown to be important in merozoite invasion by inhibition with specific antibodies and also analysis of *P. falciparum* parasites in which the gene encoding them have been disrupted [3], [4], [5], [6], [7]. This family consists of EBA-175 (MAL7P1.176), EBA-181 (JESEBL) (PFA0125c), EBL-1 (GenBank: AAD33018.1), EBA-165 (PEBL) (PFD1155w) and EBA-140 (BAEBL) (MAL13P1.60) [4], [8], [9]. These proteins belong to a larger family of proteins in *Plasmodium spp.* that includes the Duffy binding proteins (DBP) in *P. vivax* and *P. knowlesi* [9]. EBA-175 and EBA-140 bind to glycophorin A and C respectively in a sialic acid-dependent manner and are responsible for specific invasion pathways through these receptors [9], [10], [11], [12]. Two other ligand-receptor interactions requiring sialic acid are EBA-181, which binds to an unknown receptor [13] and EBL-1 to glycophorin B, an interaction of lower significance since approximately 50% of *P. falciparum* strains analysed expressed a truncated protein [14]. EBA-165 appears to be a transcribed pseudogene as the protein has not been shown to be expressed in any *P. falciparum* parasites to date [15].

The second family of proteins important for invasion of merozoites is the reticulocyte binding-like (RBP) proteins of *Plasmodium spp.* that includes the Py235 family of *P. yoelii* and the *P. vivax* RBP 1 and 2 proteins [16], [17]. These proteins have been implicated in mediating reticulocyte preference for *P. yoelii* and *P. vivax*. In *P. falciparum*, this family includes PfRh1 (PFD0110w), PfRh2a (PF13_0198), PfRh2b (MAL13P1.176), PfRh3 (PFL2520w), PfRh4 (PFD1150c) and PfRh5 (PFD1145c) [6], [7], [18], [19], [20], [21], [22], [23]. The *PfRh3* gene is a transcribed pseudogene in *P. falciparum* parasites whilst the other genes are differentially expressed and are localised to the neck of the rhoptries before merozoite invasion [6], [24]. The PfRh1, PfRh4 and PfRh5 proteins bind to specific receptors on the erythrocyte and the physical properties of these have been defined by analysis of binding and invasion into neuraminidase-, trypsin- and chymotrypsin-treated erythrocytes [7], [18], [19], [20], [22], [25], [26], [27], [28], [29]. PfRh1 binds to a neuraminidase-sensitive receptor [18], [19] whilst PfRh4 and PfRh5 bind different receptors in a sialic acid-independent manner ie. neuraminidase-resistant [25], [26], [28]. PfRh2a and PfRh2b have not been directly demonstrated to bind erythrocytes but analyses of *P. falciparum* gene knockout strains have shown that the latter protein functions in merozoite invasion [6]. In contrast, PfRh2a appears to be inactive in some strains of *P. falciparum* despite expression of the protein [6], [30]. Recently, Complement receptor 1 (CR1) has been identified as a receptor for *P. falciparum* invasion of erythrocytes [31], [32] and PfRh4 binds directly to this receptor to mediate merozoite invasion [28].

*P. falciparum* strains are able to utilise variable patterns of alternate host receptors and this provides a mechanism of phenotypic variation to evade host immune responses and circumvent the polymorphic nature of receptors on the erythrocyte surface within the human population [6]. This is mediated by differential expression and function of both the EBL and PfRh proteins [6], [25], [33], [34]. *P. falciparum* strains show a wide range of PfRh1 expression as a result of amplification of the gene and disruption of ligand expression by gene knockout results in increased function of other ligands from these families [7]. Both PfRh2a and PfRh2b are highly expressed in some *P. falciparum* lines; however, cannot be detected in others despite the presence of intact genes suggesting that they are differentially silenced [6], [35]. It has not yet been demonstrated that these ‘silenced’ genes can be activated. In contrast, PfRh4 is also silenced in some lines and activated in a small proportion of the population and these can be selected either by disruption of the *EBA-175* gene or alternatively growth of the parasites in neuraminidase-treated erythrocytes [25], [36]. These selected parasites are more dependent on the PfRh4 ligand and CR1 receptor for merozoite invasion and this ability to switch ‘invasion pathways’ provides an additional mechanism of phenotypic variation for entry into the host cell [25], [32].

Proteolytic cleavage of *P. falciparum* ligands plays an essential role in their function and fragments of the EBL and PfRh proteins are released into the supernatant during the invasion process [7], [37]. It has been demonstrated that members of the EBL and PfRh family of proteins can be cleaved in the transmembrane region by the membrane associated protease rhomboid 4 (PfROM4) (PFE0340c) which has implicated this protease in release of these proteins during invasion [29], [37], [38]. EBA-175, PfRh1 and PfRh4 appear to be cleaved by PfROM4 as the expected products are produced during invasion [29], [37]. PfRh1 and PfRh4 undergo a series of processing events during merozoite development resulting in a number of fragments and this appears to be critical for their function [29].

In this work, we show that PfRh2a and PfRh2b undergo a complex series of proteolytic cleavage events and that a defined region of the N-terminus directly binds erythrocytes. We show this region is critical to the function of these ligands as antibodies inhibit receptor binding and merozoite invasion.

## Results

### PfRh2a and PfRh2b undergo processing in the schizont stage of *P. falciparum*

**Figure 1:**
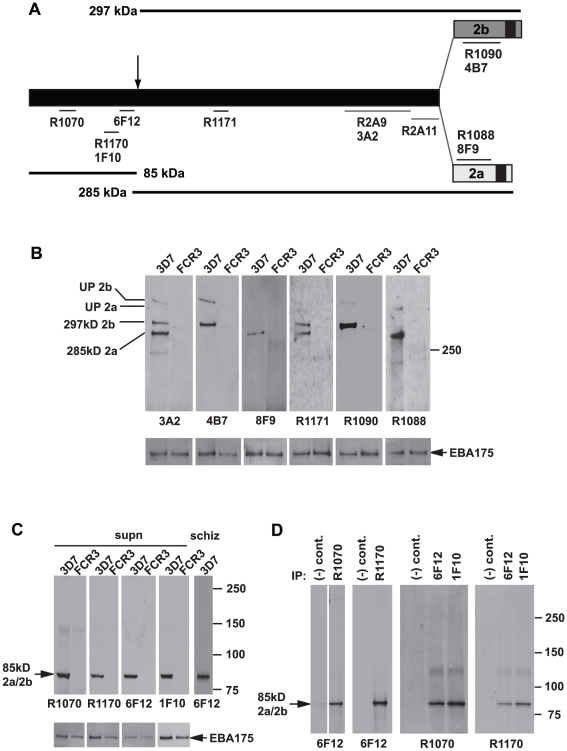
Both the PfRh2a and PfRh2b proteins are processed to release an 85 kDa product into culture supernatants. (**A**) Schematic representation of the antibodies used in this study and the approximate location of the processing event leading to a common 85 kDa fragment and unique 285 kDa PfRh2a and 297 kDa PfRh2b products. The C-terminal transmembrane domains of both the PfRh2a and PfRh2b proteins are shown. Antibodies prefaced with the letter ‘R’ are Rabbit antibodies while the other antibodies are Mouse monoclonals. (**B**) Culture supernatants contain unprocessed and large processed fragments for both PfRh2a and 2b proteins. Western blots probed with six antibodies recognising a PfRh2 common region (3A2, R1171), Rh2a-specific (8F9, R1088) and PfRh2b-specific (4B7, R1090) regions are shown. The 3A2 and R1171 antibodies detect all four PfRh2a and PfRh2b processed and unprocessed proteins in 3D7 supernatants. The 4B7 and R1090 antibodies detect the processed 297 and unprocessed 382 kDa PfRh2b proteins. The 8F9 and R1088 antibodies detect the processed 285 and unprocessed 370 kDa PfRh2a proteins. Apart from weak reactivity with both unprocessed and processed PfRh2b proteins with the 4B7 and R1090 antibodies, no proteins from FCR3 supernatants were detected. As a loading control, EBA-175 antibodies were used to probe the same blots. (**C**) Only N-terminal antibodies detect the 85 kDa processed product both in culture supernatants and schizonts. Four different N-terminal antibodies detect the 85 kDa processed product in 3D7-derived supernatants and schizonts but not in FCR3 supernatants. The same blots were reprobed with EBA-175 antibodies as a loading control. (**D**) Four different N-terminal antibodies immunoprecipitate the 85 kDa product. 3D7 culture supernatants were immunoprecipitated with all four N-terminal antibodies. Immunoprecipitates derived from rabbit antibodies were probed with mouse antibody 6F12 and immunoprecipitates derived from mouse antibodies were probed with both rabbit antibodies.

PfRh2a and PfRh2b proteins are identical for most of the N-terminus and completely diverge in sequence 42 and 54 kDa respectively from the C-terminus (Figure 1A) [19], [20]. To determine the role of proteolytic cleavage in these proteins a panel of six recombinant proteins were produced that spanned PfRh2a and PfRh2b and polyclonal and monoclonal antibodies raised (Figure 1A). The six recombinant proteins were in addition to two other fusion proteins that had previously been used to generate the rabbit antibodies, R2A9 and R2A11 (Figure 1A) [20]. Polyclonal (R1090, PfRh2b-specific and R1088, PfRh2a-specific) and monoclonal antibodies (4B7, PfRh2b specific and 8F9, PfRh2a specific) were made to the unique regions of PfRh2a and PfRh2b. Additionally, polyclonal (R1070, R1170 and R1171) and monoclonal antibodies (6F12, 1F10, 3A2) were derived to the common N-terminus of both proteins (Figure 1A). The specificity of these antibodies was confirmed by immunoblot using parasite lines 3D7 and FCR3, in which the former expresses PfRh2a and PfRh2b whilst the latter does not (Figure 1B) [7]. The monoclonal antibody 3A2 bound to protein bands corresponding to that expected for both PfRh2a and PfRh2b which were absent in FCR3 parasites. In contrast, the 4B7 monoclonal antibody bound to the protein bands corresponding to PfRh2b, which were absent in FCR3. The 8F9 monoclonal antibody bound specifically to bands expected for PfRh2a expressed in 3D7. The R1171 rabbit polyclonal bound to both major PfRh2a and b protein bands. The R1090 and R1088 antibodies were specific to PfRh2b and PfR2a respectively. Therefore the monoclonal antibody 4B7 and the polyclonal antibody R1090 are specific for PfRh2b whilst the monoclonal antibody 8F9 and polyclonal antibody R1088 reacts specifically with PfRh2a. In contrast, the monoclonal antibody 3A2 and polyclonal rabbit antibodies R1171 react with both PfRh2a and PfRh2b.

Whilst the large PfRh2a and PfRh2b proteins were detected previously [6], [7], [18], [19], [20], [21], [22], [23], antibodies to the N-terminus have not been available (Figure 1C). Immunoblots of 3D7 culture supernatants unexpectedly identified an 85 kDa protein using the four antibodies to different regions of this domain (R1070, R1170 and monoclonals 6F12 and 1F10) (Figure 1A and 1C). The same sized 85 kDa protein was also detected in purified 3D7 schizonts, suggesting that this processing event occurred before rupture and release of merozoites for invasion. To confirm that R1070, R1170, 1F10 and 6F12 antibodies were detecting the same 85 kDa polypeptide from the PfRh2a and PfRh2b proteins we immunoprecipitated with each and probed immunoblots with the other antibodies (Figure 1D). The results show that the four antibodies can specifically immunoprecipitate the 85 kDa processed PfRh2a/2b fragment and that this can be detected by immunoblot using the other antibodies. Therefore the full length PfRh2a (370 kDa) and PfRh2b (382 kDa) proteins are processed into two major peptides, during late schizont development, of approximately 85/285 kDa for PfRh2a and 85/297 kDa for PfRh2b.

### PfRh2a and PfRh2b processing occurs in a post-Golgi compartment

**Figure 2:**
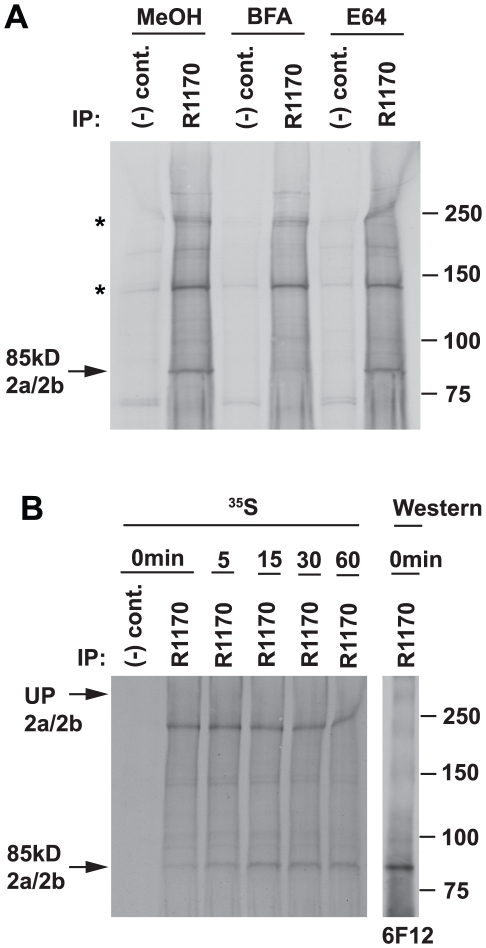
Processing of the 85 kDa fragment is brefeldin A-sensitive. (**A**) Sensitivity of the production of the 85 kDa fragment to BFA and E64. Synchronised 3D7 schizont cultures were treated with BFA at 5 ug/ml (methanol as control) or E64 at 10 µM (water as control) for 1 h before resuspending in methionine/cysteine-free medium and [^35^S]methionine/cysteine for a further 1 h with inhibitors as before. Labelled parasites were then lysed and normal rabbit serum [(−) control] or R1170 anti-85 kDa fragment antibody used for immunoprecipitations. The 85 kDa band is arrowed while two cross-reacting bands at ∼140 and ∼220 kDa are asterisked. (**B**) The level of the 85 kDa fragment peaks at a 15 min chase time. 3D7 schizonts were labelled with [^35^S]methionine/cysteine for 10 min, then chased for 0, 5, 15, 30 and 60 min. Samples were immunoprecipitated with normal rabbit serum or R1170. The right panel shows 0 min samples probed with an antibody to the 85 kDa fragment (6F12).

Previously, it has been shown that processing of PfRh2a and PfRh2b was brefeldin A (BFA) sensitive implying that a protease cleavage event occurred post-Golgi in the schizont [24]. To show that cleavage and release of the 85 kDa polypeptide derived from the full-length PfRh2a and PfRh2b proteins occurred after trafficking through the Golgi, we radiolabelled 3D7 schizonts in the presence or absence of BFA and immunoprecipitated with an antibody (R1170) to the 85 kDa fragment (Figure 2 A). BFA blocked appearance of the N-terminal 85 kDa polypeptide showing that this fragment was derived by processing from the full-length proteins, and also suggests this occurs at the schizont stage before merozoite egress (Figure 2A). To confirm that processing occurs prior to merozoite egress, 3D7 parasites were radiolabelled in the presence or absence of the cysteine protease inhibitor E64, an inhibitor of schizont rupture [39], [40]. These experiments showed that E64 had no effect on cleavage of the PfRh2a/b proteins to the 85 kDa polypeptide consistent with this processing event occurring in schizonts prior to merozoite egress (Figure 2A).

To further confirm that the 85 kDa fragment was produced by processing of the full-length PfRh2a and PfRh2b proteins we used pulse chase experiments followed by immunoprecipitation with an N-terminal antibody (Figure 2B). Synchronized 3D7 schizonts were radiolabelled with [^35^S]methionine/cysteine and chased with the same nonradioactive amino acids. Samples were taken following the commencement of cold chase and immunoprecipitated with R1170, an antibody to the 85 kDa fragment. At the beginning of the chase there was little of the 85 kDa fragment, but this accumulated over the time points peaking at 15 minutes (Figure 2B). A protein band of approximately 380 kDa, that co-migrated with the large unprocessed PfRh2a/b proteins (Figure 2B), was very faintly visible that decreased in its accumulation concomitant with the increase in the 85 kDa band suggesting it is the precursor protein. However, this large molecular weight band was not heavily labelled consistent with the very low levels of the full-length PfRh2a/b proteins observed in immunoblots (Figure 1B). The identity of the radiolabelled chased 85 kDa fragment was confirmed by Western blot with the 6F12 antibody (Figure 2B). Taken together these data show that the full-length PfRh2a and PfRh2b proteins are proteolytically cleaved in the late schizont stage to produce an N-terminal 85 kDa fragment.

### The 85 kDa processed form of PfRh2a and PfRh2b co-localises and forms a complex with the corresponding C-terminal regions

**Figure 3:**
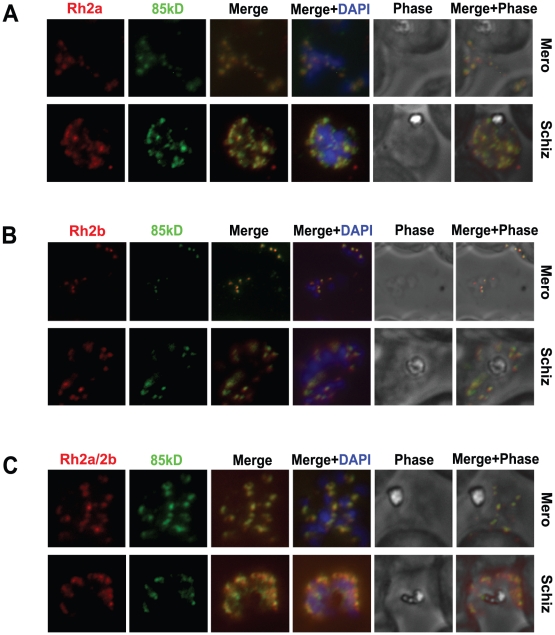
The 85 kDa processed form of PfRh2a and PfRh2b colocalises with the corresponding C-terminal regions in both merozoite and schizont stages. (**A**) The 85 kDa product co-localises with the C-terminal PfRh2a product. Free merozoites or schizonts of the 3D7 parasite were dual stained with 8F9 anti-PfRh2a and R1170 anti-85 kDa antibodies. Both merozoites and schizonts give an apical staining pattern with both antibodies, indicating co-localisation. Nuclei were stained with DAPI. (**B**) The 85 kDa product co-localises with the C-terminal PfRh2b product. Parasites were dual stained with 4B7 anti-PfRh2b and R1170 anti-85 kDa antibodies. As for (A), both antibodies give a co-localised apical staining pattern. (**C**) The 85 kDa product co-localises with both the PfRh2a and PfRh2b C-terminal products. Parasites were dual stained with 3A2 anti-PfRh2a/2b and R1170 anti-85 kDa antibodies. As for (A), both antibodies give a co-localised apical staining pattern.

To determine if the 85 kDa polypeptide cleaved from the PfRh2a and PfRh2b proteins in schizont stages co-localised with the C-terminal fragments of each protein we used synchronised parasites for immunofluorescence experiments. The PfRh2a specific monoclonal antibody 8F9 (detects the C-terminal polypeptide) and the R1170 rabbit antibody (detects the N-terminal 85 kDa domain) both showed an apical pattern with strong co-localisation (Figure 3A). To determine if the 85 kDa fragment also co-localised with the 297 kDa C-terminal PfRh2b polypeptide parasites were dual stained with the monoclonal antibody 4B7 (detects the C-terminal PfRh2b protein) and R1170 (detects the N-terminal 85 kDa of PfRh2a and PfRh2b) rabbit antibody. Both antibodies showed a staining pattern of the apical end in merozoites with clear co-localisation (Figure 3B). This suggested that the PfRh2a and PfRh2b cleaved polypeptides had the same subcellular co-localisation in schizont and merozoite stages. To confirm this parasites were dual stained with an antibody recognising the PfRh2a and PfRh2b C-terminal polypeptides and the 85 kDa N-terminal domain using R1170 rabbit antibodies. Both antibodies showed co-localisation in schizont and merozoite stages. (Figure 3C). These results have confirmed that following cleavage of PfRh2a and PfRh2b the 85 kDa N-terminal fragment and the large C-terminal processed polypeptides co-localise in schizont and merozoite stages of *P. falciparum* consistent with them existing as a complex (Figure 3C).

**Figure 4:**
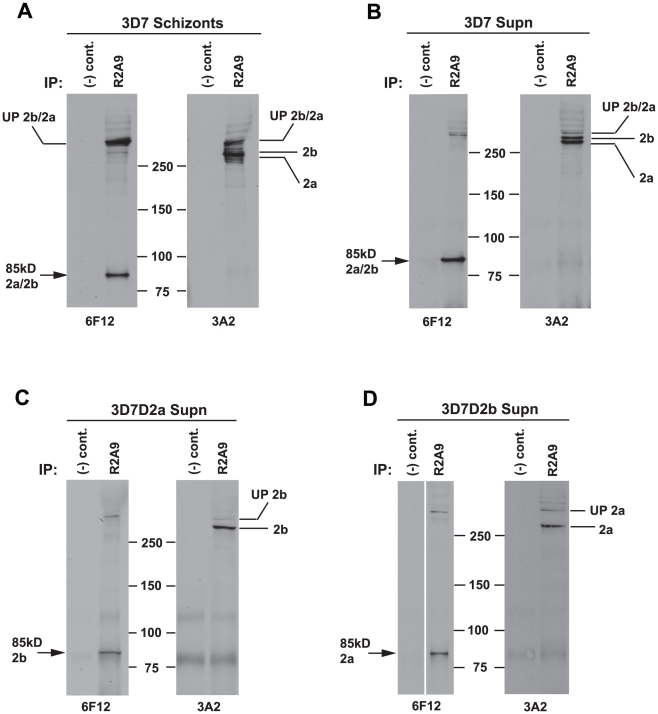
Both PfRh2a and PfRh2b proteins form a complex with the 85 kDa processed product. (**A**) Proteins from synchronised 3D7 schizont stage parasites were immunoprecipitated with either R2A9 Abs or normal rabbit serum [(−) control]. Immunoprecipitated proteins were separated by SDS-PAGE and probed with either 6F12 or 3A2 antibodies. The left panel shows that the R2A9 immunoprecipitate also brings down the 85 kDa processed product as detected with the 6F12 antibody, indicating that in 3D7 schizonts, the 85 kDa product is complexed with both the 285 kDa PfRh2a protein and the 297 kDa PfRh2b protein, as shown in the Right panel. (**B**) Proteins from 3D7 culture supernatants were immunoprecipitated, separated by SDS-PAGE and probed as in (A) above. The result shows that the 285/85 kDa PfRh2a and the 297/85 kDa PfRh2b complexes still remain in supernatant following invasion. (**C–D**) In order to confirm that both PfRh2a and PfRh2b were capable of complex formation, proteins from culture supernatants of both 3D7Δ2a and 3D7Δ2b parasites were immunoprecipitated as before. The results show that both PfRh2b (C) and PfRh2a (D) can form a complex with the 85 kDa processed product.

To determine if the 85 kDa N-terminal region of PfRh2a and PfRh2b formed a complex with the corresponding C-terminal regions of each protein we used 3D7 schizont stage parasites in immunoprecipitation experiments with the R2A9 polyclonal antibodies (recognises C-terminal PfRh2a and PfRh2b domain) (Figure 4). The precipitated proteins were detected in immunoblots using the monoclonal 6F12 that recognised the N-terminal 85 kDa domain as well as the full-length PfRh2a and PfRh2b proteins (Figure 4A). The same immunoprecipitate probed with the monoclonal 3A2 recognised the unprocessed and C-terminal processed forms of PfRh2a and PfRh2b. Similar results were obtained in immunoprecipitation experiments with culture supernatants (Figure 4B). We did similar experiments with 3D7Δ2a and 3D7Δ2b, parasites that lack expression of PfRh2a or PfRh2b respectively, to determine if both proteins could form a complex after N-terminal processing [6]. This confirmed that PfRh2a and PfRh2b are both processed to yield the 85 kDa N-terminal fragment and that this can form a complex with either the PfRh2a or PfRh2b C-terminal domain and that these complexes are shed into the culture supernatant during merozoite invasion of red blood cells (Figure 4C and D).

Both the 85 kDa and 285/297 kDa C-terminal products of Rh2a/Rh2b have multiple cysteine residues and to determine if the complex formed between the N-terminal and C-terminal polypeptides are held together through disulphide bonds we used SDS-PAGE under non-reducing conditions and as a comparison also under reduced conditions (Figure S1). Proteins from culture supernatants were probed with antibodies that would detect the PfRh2a and PfRh2b 85, 285 and 297 kDa polypeptides. These proteins migrated at an identical size compared to that observed under reducing conditions indicating the complex did not involve disulphide bonds (Figure S1).

### PfRh2a is C-terminally processed during merozoite invasion

**Figure 5:**
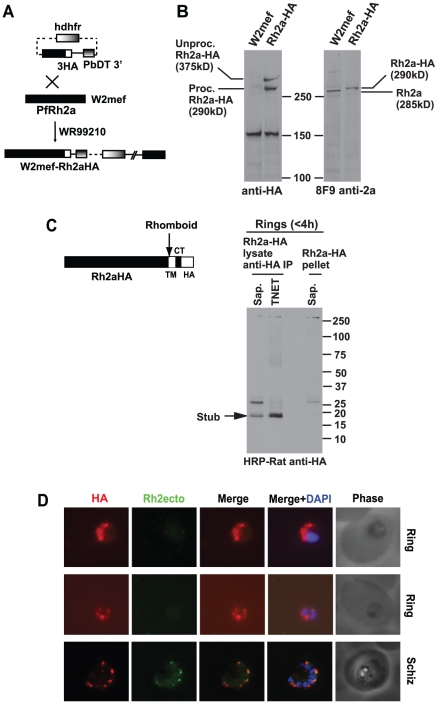
C-terminal tagging of the PfRh2a protein in *P. falciparum* by transfection. (**A**) The plasmid for tagging PfRh2a and PfRh2b with 3×HA epitopes by 3′ crossover recombination is shown. The human dihydrofolate reductase-thymidylate synthase (*hdhfr*) gene encodes resistance to WR99210. The whole cassette that includes a promoter and terminator is labelled as *hdhfr*. The *P.berghei dhfr* 3′ terminator region (PbDT 3′) served as the terminator for the gene being tagged. (**B**) *Pfrh2a* is tagged with 3HA. Proteins from schizont stages of the W2mef parasite or the line expressing tagged PfRh2a (Rh2aHA) were separated by SDS-PAGE then probed with either anti-HA Abs (Left panel) or the 8F9 antibody to the PfRh2a protein (Right panel). The anti-HA antibody detect two high molecular weight proteins specifically in the tagged line, a processed protein at ∼295 kDa and the unprocessed protein at ∼380 kDa. The protein detected at ∼150 kDa is a cross-reacting protein found also in the untagged wild-type parasite. (**C**) PfRh2a is C-terminally cleaved at invasion and the resulting stub carried into the Ring. The schematic diagram shows the C-terminal cleavage, most likely due to a rhomboid protease, occurring within the transmembrane (TM) region of Rh2aHA parasites. The HA-tagged cytoplasmic tail (CT) is also shown. A synchronised mixed schizont/Ring-stage culture, containing Rings <4 h old, was sorbitol-synchronised to remove schizonts. The young Rings were then lysed either with saponin or TNET, spun down, and both supernatant and pellet fractions collected. HA-containing proteins from both supernatant fractions were immunoprecipitated with anti-HA Abs. Immunoprecipitated proteins together with proteins from the saponin and TNET pellets were separated by SDS-PAGE, blotted, then probed with anti-HA antibodies. The C-terminally processed tail of ∼18 kDa was detected in both the saponin and TNET extracted ring fraction but not in the saponin-lysed pellet fraction. A cross-reacting protein at ∼25 kDa was seen only in the saponin fractions. (**D**) The HA-tagged stub is carried into ring stages. Young rings less than 4 h old and schizonts of the Rh2aHA line were dual stained with 12CA5 anti-HA (Roche, Germany) and a common region antibody (R2A11, Figure 1). The PfRh2a/b ecto-domain is not carried into rings, while the HA-tagged stub is carried into rings. Nuclei were stained with DAPI.

In order to follow the processing events before and after merozoite invasion we attempted to tag the 3′ end of the *Pfrh2a* and *Pfrh2b* genes with haemagglutinin (HA) epitopes by single crossover homologous recombination (Figure 5A) [41]. Unfortunately, we were unable to select parasites in which the plasmid had inserted into the *Pfrh2b* gene; however, this was successful for *Pfrh2a* by transfection of W2mef parasites to derive the parasite line W2mefRh2a-HA. Successful tagging was shown by immunoblots using anti-HA antibodies that detected two bands corresponding to PfRh2a in the transfected line but not the parental parasite (Figure 5B, left panel). This was confirmed using the PfRh2a specific 8F9 monoclonal antibody that identified the protein in W2mef, and an approximately 5 kDa larger protein, corresponding to the epitope tag, in the W2mefRh2a-HA transfected line (Figure 5B, right panel). Despite the small size of the tag, its very acidic characteristics (pK_i_ = 3.80), may contribute to it causing a significant mobility difference on SDS-PAGE between the untagged and tagged versions of PfRh2a.

The ligand EBA-175 is cleaved by the protease PfROM4 at the C-terminus in the transmembrane domain and we have demonstrated that PfRh1 and PfRh4 are similarly cleaved during merozoite invasion resulting in release of these processed proteins into the supernatant [29], [37]. To test if PfRh2a was cleaved we purified ring stages less than 4 h post invasion to determine if the transmembrane and cytoplasmic tail of PfRh2a, potentially released by PfROM4, could be detected using anti-HA antibodies (Figure 5C). Immunoprecipitation of saponin or TNET solubilised proteins using mouse anti-HA antibodies followed by detection with rat anti-HA antibodies recognised a band of ∼17 kDa (Figure 5C). While the calculated size of the protein stub following rhomboid cleavage within the transmembrane domain is 11 kDa, the highly acidic nature of this stub (pK_i_ = 3.77) may cause it to migrate aberrantly on SDS-PAGE. Hence this indicated that the tagged PfRh2a protein was C-terminally cleaved during merozoite invasion removing a stub migrating at ∼17 kDa, corresponding in size to the transmembrane, cytoplasmic tail and HA_3_ epitope tag, consistent with the function of a PfROM protease to release the ectodomain into the supernatant. In addition, a small proportion (<5%) of unprocessed PfRh2a, migrating at over 250 kDa, was observed indicating that proteolytic cleavage of the cytoplasmic tail was highly efficient during merozoite invasion (Figure 5C). To confirm that the HA-tagged PfRh2a C-terminal stub was carried into the ring stage of the parasite, synchronous PfRh2aHA parasites containing late schizont and early ring forms were dual stained with anti-HA and PfRh2a/b common region antibodies (Figure 5D). As expected the developing merozoites in schizont stages showed apical staining with both anti-HA and anti-PfRh2a/b ectodomain antibodies. Following invasion the ring stage parasites reacted with anti-HA antibodies indicating that the HA-tagged stub was carried into ring stages, while the PfRh2a/b ectodomain was not, but instead was presumably released into the culture supernatant. This was consistent with PfROM4 cleavage of PfRh2a, and by inference PfRh2b, during merozoite invasion that would result in shedding of the 285 kDa PfRh2a and 297 kDa PfRh2b ectodomains and the 85 kDa N-terminal region into the culture supernatant with the remaining cleaved stub carried into the ring stage after invasion of the red blood cell (Figure 1B). This result is consistent with previous data showing that the PfRh2a and b putative recognition sequences for PfROM4 can be cleaved by this protease when expressed in mammalian cells [29], [37], [38].

### The PfRh2a/b C-terminal fragment localises to the rhoptry neck and moving junction of merozoites

**Figure 6:**
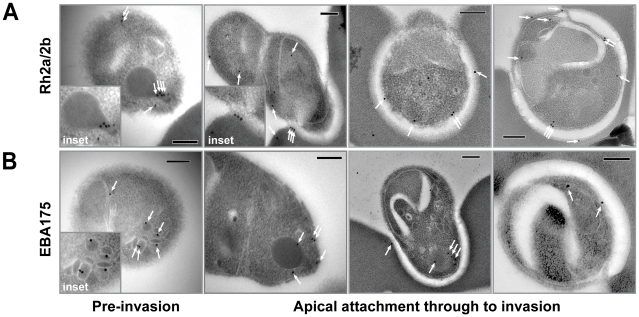
Immuno-electron microscopy localisation of PfRh2a and PfRh2b during merozoite invasion of human erythrocytes. (**A**) The PfRh2a/2b proteins localise to the rhoptry neck and moving junction of merozoites. Transmission electron micrographs of pre- and mid-invasion merozoites showing PfRh2a/b-specific immunogold labelling (white arrows). At the pre-invasion stage, PfRh2a/b localises to the rhoptry neck (inset), while during mid-invasion, PfRh2a/b localises both to the moving junction and to the merozoite surface within the invasion pit. Scale bar = 0.2 µm. (**B**) The EBA-175 protein localises to the micronemes and apical tip of merozoites. At the pre-invasion stage, EBA-175 is located within micronemes (inset), while during mid-invasion, EBA-175 can be located either at the apical tip or within the invasion pit. Scale bar = 0.2 µm.

We have previously localised PfRh2a and PfRh2b to the neck of the rhoptries in merozoites by immunoelectron microscopy [6]. However, it has not been technically possible to purify viable *P. falciparum* merozoites in sufficient quantities to allow the localisation of proteins during invasion of erythrocytes by immunoelectron microscopy. Recently, a new method has been developed for purification of invasive merozoites and we used this to superinfect erythrocytes to trap *P. falciparum* parasites during invasion for the localisation of PfRh2a and PfRh2b by immuno-electron microscopy with antibodies to the conserved common domain of both proteins. As we have described previously, PfRh2a/b was predominantly retained within the rhoptry neck before active invasion (Figure 6C, inset) [6]. After attachment, the results suggested movement of PfRh2a/b from within the rhoptry neck to the merozoite surface in some parasites, predominantly anterior to the tight junction within the invasion pit. In other instances, evidence for immunogold labelling at the tight junction was observed (Figure 6C), supporting recent immunofluorescence data for another PfRh family member, PfRh1 [6]. For a comparison we also labelled free merozoites with EBA-175 antibodies as the PfRh and EBL protein families appear to have overlapping functions in erythrocyte invasion [[25], [42]. The antibodies to EBA-175 displayed a micronemal localisation (Figure 6D) as has been shown previously [43]. From attachment through to invasion localisation was difficult to follow, but suggested that surface-released EBA-175 capped the apical tip, with labelling seen in the invasion pit of some parasites caught mid invasion (Figure 6D). A proportion of EBA-175 labelling, however, remained within the apex of the merozoite, even after initiation of invasion. The overlap in function between EBL and PfRh proteins during invasion shown previously, is consistent with the images shown here suggesting that these proteins play an important role at or close to the tight junction during merozoite invasion [25], [42].

### The 85 kDa cleaved PfRh2a and PfRh2b protein binds erythrocytes

**Figure 7:**
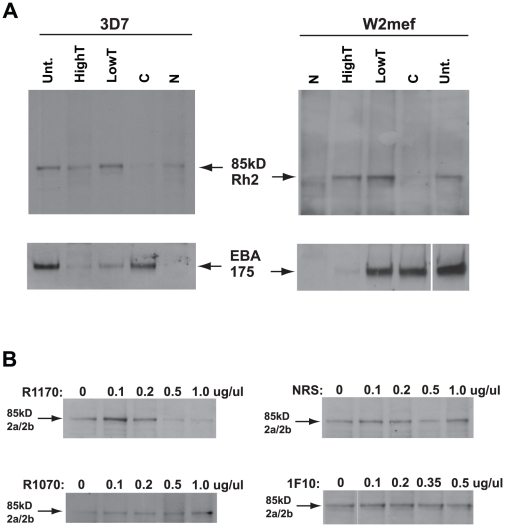
The PfRh2a/b 85 kDa processed product binds erythrocytes and binding is inhibited by antibodies to the binding domain. (**A**) Culture supernatant from both 3D7 and W2mef parasites was bound to untreated (Unt.), Low trypsin (0.067 mg/ml), High Trypsin (1 mg/ml), neuraminidase (N) or chymotrypsin-treated (C) erythrocytes. Bound proteins were eluted with 1.5 M NaCl, separated on SDS-PAGE gels, Western blotted and probed with an antibody to the 85 kDa product (6F12) and an antibody to EBA-175. PfRh2a/b binding to erythrocytes was sensitive to neuraminidase and chymotrypsin, but resistant to both Low and High Trypsin concentrations. The input culture supernatant is labelled as ‘supn’. The lower panels probed with anti-EBA-175 Abs were included as a control for erythrocyte enzyme treatments as it is known that EBA-175 binding is neuraminidase sensitive, partially trypsin sensitive, but chymotrypsin resistant. (**B**) R1170 antibodies block native PfRh2a/b binding. Protein G-purified R1070, R1170 or normal rabbit serum antibodies at final concentrations from 0.1 to 1.0 µg/µl or the 1F10 monoclonal antibody at final concentrations from 0.1 to 0.5 µg/µl were preincubated with 3D7 culture supernatant before adding Untreated erythrocytes. Bound proteins were eluted with 1.5 M NaCl, separated on SDS-PAGE gels, blotted and probed with an antibody (6F12) to the 85 kDa PfRh2a/b binding domain.

It has been suggested previously that the large PfRh2a and PfRh2b proteins shed into culture supernatants did not bind red blood cells and additional experiments using new antibody reagents, generated in this study, was consistent with these results (Figure S2) [6]. However, PfRh2b was functional in 3D7 parasites as it was possible to inhibit invasion with antibodies to this protein suggesting it interacted either directly or indirectly with a trypsin- and neuraminidase-resistant erythrocyte receptor. Identification of an 85 kDa fragment from both PfRh2a and PfRh2b raised the possibility that this contained the erythrocyte binding domain. In order to test this possibility we performed erythrocyte-binding assays using culture supernatant from 3D7 and W2mef parasites (Figure 7A). Using the anti-PfRh2a/b monoclonal 6F12 we detected the 85 kDa protein from both 3D7 and W2mef after it was eluted from red blood cells. This binding was trypsin-resistant and partially chymotrypsin- and neuraminidase-sensitive which is similar but not identical to the physical properties of the host receptor determined previously by analysing PfRh2b parasites in which the gene had been disrupted as well as inhibition of function with specific antibodies for invasion into enzyme-treated erythrocytes [6]. Taken together these results show that the 85 kDa PfRh2a and PfRh2b polypeptide contains the binding site for human erythrocytes.

To confirm that the 85 kDa PfRh2a and 2b protein was able to bind erythrocytes, we tested antibodies to this region (Figure 1) for their ability to block binding of the 85 kDa protein to erythrocytes. Rabbit antibodies (R1070 and R1170) and both the 6F12 and 1F10 monoclonals were used at concentrations from 0.1 to 1.0 ug/ul and only R1170 antibody efficiently blocked binding of native PfRh2a/b to erythrocytes (Figure 7B). Normal rabbit serum, polyclonal antibodies to other regions of the 85 kDa domain (R1070, Figure 7B) and the monoclonal antibody 1F10 (Figure 7B) and 6F12 (data not shown) did not block binding. This suggested that the binding region was located towards the C-terminus of the 85 kDa polypeptide (Figure 1).

### The binding domain of the 85 kDa PfRh2a and b proteins

**Figure 8:**
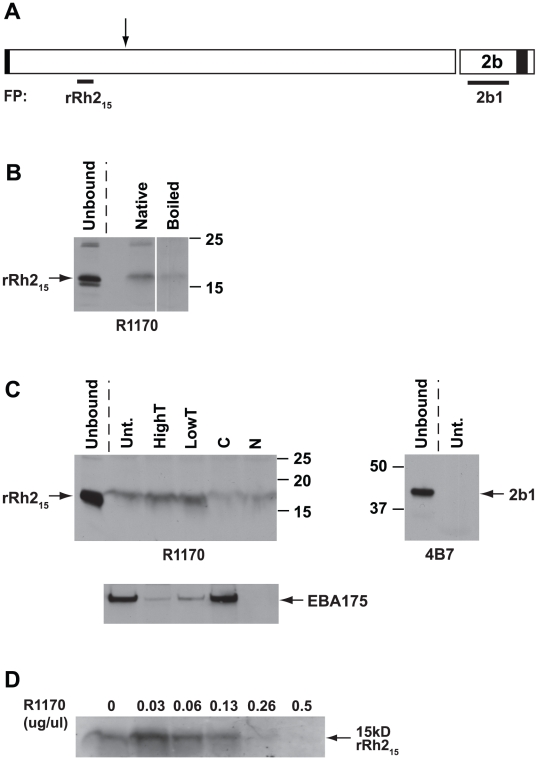
Recombinant rRh2_15_ binds erythrocytes. (**A**) Schematic diagram of the PfRh2a/b protein showing the location of the rRh2_15_ and 2b1 fusion proteins. The rRh2_15_ is located within the 85 kDa binding domain of PfRh2a/b. The processing event leading to the 85 kDa product is indicated by the arrow. The 2b1 fusion protein is from a PfRh2b unique region at the C-terminus of the protein. The regions of the protein in black at the N and C-termini represent the signal sequence and transmembrane domains respectively. (**B**) 1 µg rRh2_15_ (native) or 1 µg denatured rRh2_15_ (boiled) were bound to untreated erythrocytes. Bound proteins were eluted with 1.5 M NaCl, separated on SDS-PAGE gels, blotted and probed with an antibody (R1170) to the rRh2_15_ fusion protein. Unbound proteins removed from the Untreated erythrocytes are also shown. (**C**) 1 µg rRh2_15_ was bound to untreated (Unt.), Low trypsin (0.067 mg/ml), High Trypsin (1 mg/ml), neuraminidase (N) or chymotrypsin-treated (C) erythrocytes. Bound proteins were eluted, separated and probed as in B above. In the lower panel, both enzyme-treated and untreated erythrocytes were also probed with an antibody to EBA-175 to ensure the enzyme treatments had been successful. Recombinant rRh2_15_ binding to erythrocytes was partially sensitive to neuraminidase and chymotrypsin, but resistant to both Low and High Trypsin concentrations. Unbound proteins removed from the Untreated erythrocytes are also shown. The 2b1 fusion protein was bound to untreated erythrocytes. Bound proteins were eluted with 1.5 M NaCl, separated on SDS-PAGE gels, Western blotted and probed with the 4B7 antibody raised to the 2b1 fusion protein. The 2b1 fusion protein showed no binding to Untreated erythrocytes but was clearly present in the Unbound fraction. (**D**) R1170 antibodies block binding of rRh2_15_ to erythrocytes. Protein G-purified R1170 antibodies at final concentrations from 0.03 to 0.5 µg/µl were preincubated with 0.5 µg rRh2_15_ fusion protein before adding Untreated erythrocytes. Bound proteins were eluted with 1.5 M NaCl, separated on SDS-PAGE gels, Western blotted and probed with Protein G-purified R1170.

In order to define the receptor binding region within the 85 kDa PfRh2a and b proteins we made a recombinant hexa-His tagged protein of 15 kDa corresponding to amino acids 446 to 557 (rRh2_15_). This protein fragment was located close to similar regions of PfRh1 and PfRh4 that have been shown to bind erythrocytes (Figure S3 and S4) [26], [27], [28], [32]. Rabbit antibodies (R1170) made to rRh2_15_ that block binding of native PfRh2a and b to erythrocytes have already been described above (Figure 7B). The rRh2_15_ fusion protein (Figure S5) bound to erythrocytes whereas the 2b1 protein (Figure S5) from the C-terminal region of PfRh2b showed no detectable binding (Figure 8A, 8B and 8C). Additionally, we tested binding of a number of other recombinant proteins corresponding to different regions of the PfRh2a, PfRh2b and PfRh1 proteins and none of these regions showed binding to red blood cells (Figure S6). The specificity of binding of the rRh2_15_ fragment was shown by heat denaturing the fusion protein and binding to erythrocytes (Figure 8B). Only the native fragment bound erythrocytes. The rRh2_15_ erythrocyte binding was resistant to trypsin treatment but partially sensitive to chymotrypsin and neuraminidase treatment, a pattern of binding we observed for the *P. falciparum* expressed 85 kDa protein from culture supernatants. The enzyme-treated erythrocytes were also used in binding assays with parasite culture supernatants and the eluted proteins probed with EBA-175 antibodies to ensure that the enzyme treatments had been successful. To show that binding of rRh2_15_ to erythrocytes was specific we showed that Protein G-purified R1170 antibodies blocked binding (Figure 8D). The R1170 antibodies inhibited binding of rRh2_15_ in a dose-dependent manner with almost full blocking at an antibody concentration of 0.26 mg/ml. (Figure 8D). Therefore the erythrocyte-binding domain of PfRh2a and b is located within the region defined by the 15 kDa rRh2_15_ recombinant protein (Figure 8).

### Antibodies to the PfRh2a/b binding site inhibit merozoite invasion

**Figure 9:**
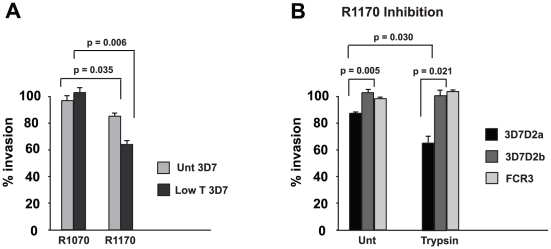
Antibodies to rRh2_15_ block merozoite invasion. (**A**) R1170 antibodies to rRh2_15_ block invasion of both untreated and Low trypsin-treated erythrocytes. Protein G-purified IgG at 2 mg/ml final concentration from both R1070 and R1170 prebleeds and kill bleed sera were added to 3D7 parasites at the trophozoite stage together with target erythrocytes that were untreated or Low trypsin (0.067 mg/ml)-treated. Following reinvasion in the presence of antibodies, cultures were continued to the trophozoite stage, when parasite numbers were determined in order to see the effect of antibodies on invasion. Percent invasion in the absence of antibodies was adjusted to 100% invasion. Experiments were done at least twice in triplicate. Error bars show the standard error of the mean. P-values were calculated using the Students t-test. (**B**) Antibodies to rRh2_15_ block invasion mediated by PfRh2b but not PfRh2a in 3D7 parasites. Protein G-purified IgG from R1170 serum at 2 mg/ml final concentration was added to 3D7Δ2a (express PfRh2b only), 3D7Δ2b (express PfRh2a only) and FCR3 (express neither PfRh2a nor PfRh2b) parasites at the trophozoite stage together with target erythrocytes that were untreated or treated with 0.03 mg/ml Trypsin. Other details of the experiments were the same as in (A) above.

Previously, it has been shown that antibodies to the C-terminus of the PfRh2a and 2b common region inhibit merozoite invasion and this confirmed that PfRh2b plays a direct role in this process [6]. To determine if antibodies to rRh2_15_ (R1170) inhibit invasion we tested them in growth inhibition assays with normal and trypsin-treated erythrocytes. The anti-rRh2_15_ antibodies showed approximately 18% inhibition of 3D7 invasion into normal erythrocytes compared to no inhibition for antibodies (R1070) to a second fusion protein away from the receptor binding site and this inhibition was increased for trypsin-treated cells to 38% (Figure 9A). The enhancement of inhibition occurred as a result of removal of trypsin-sensitive receptors from erythrocytes thus limiting those available. The PfRh2a/b erythrocyte receptor is trypsin-resistant and removal of other receptors by this treatment increased the potency of these inhibitory antibodies [6]. To show that the inhibitory effect was specific and also to determine if it was acting on the function of both PfRh2a and PfRh2b we used the *P. falciparum* lines in which each gene had been specifically disrupted (3D7Δ2a and 3D7Δ2b) or lacked expression of these proteins (FCR3) [6] (Figure 9B). For untreated erythrocytes anti-rRh2_15_ antibodies inhibited growth of 3D7Δ2a (which lacks expression of PfRh2a) at about the same level as for, the 3D7 parent (Figure 9A and B) and this was enhanced for both parasites when using trypsin-treated erythrocytes. In contrast, the *P. falciparum* lines 3D7Δ2b (lacks expression of PfRh2b) and FCR3 (lacks expression of PfRh2a and may express very low levels of PfRh2b) were not inhibited (Figure 9B). Therefore the anti-rRh2_15_ antibodies to the receptor-binding site directly inhibit only PfRh2b function but not PfRh2a function, confirming that PfRh2a was not functional in 3D7 [6], [7], [18], [19], [20], [21], [22], [23].

## Discussion

Invasion of host cells by *Plasmodium spp.* requires specific ligand-receptor interactions to identify the appropriate target followed by activation of the entry process (see for review [1]). Different *P. falciparum* strains utilise alternative host receptors for invasion of red blood cells by merozoites that provides a mechanism of phenotypic variation for evasion of host immune responses and a means to circumvent the polymorphic nature of the erythrocyte in the human population [6]. The PfRh family of proteins are key players in this process and some have been shown to bind to red blood cells and function directly in merozoite invasion [6], [7], [18], [19], [20], [21], [22], [23]. PfRh2a and PfRh2b are important members and they undergo a complex series of protease cleavage events that is critical for their function in invasion. We have shown that both proteins bind directly to erythrocytes and have defined the region involved in receptor binding. Antibodies to the receptor-binding region can directly inhibit merozoite invasion by blocking interaction with the host receptor demonstrating the important function of this domain.

**Figure 10:**
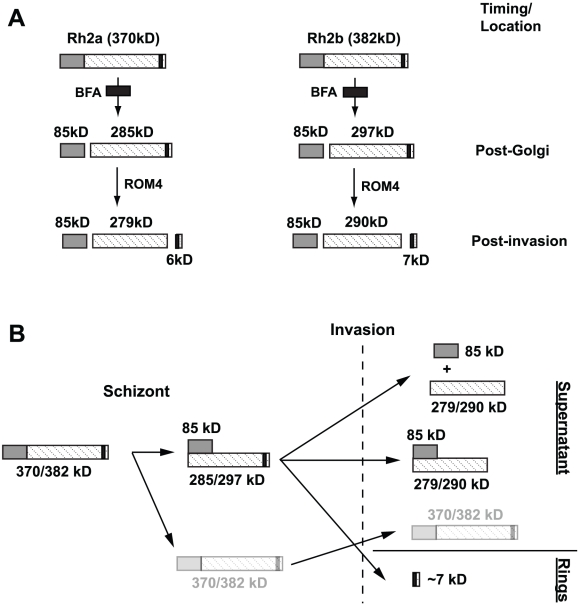
Schematic diagram illustrating post-Golgi and rhomboid processing of PfRh2a/2b proteins. (**A**) Full-length PfRh2a/2b proteins are initially processed in a BFA-sensitive post-Golgi compartment to produce the 85 kDa fragment. The proteins are then transported to the apical tip, where following invasion, processing within the transmembrane domain, most likely by PfROM4, occurs. (**B**) Schematic diagram illustrating complex formation and the fate of various PfRh2a/2b processed products. Full-length PfRh2a and PfRh2b proteins are predicted to be 370 and 382 kDa respectively. Cleavage to produce the 85 kDa product and its subsequent complexing with the transmembrane-containing PfRh2a and PfRh2b products, which occurs at the schizont stage, is shown. Following invasion, C-terminal rhomboid processing results in both complexed and uncomplexed forms being shed into culture supernatant while the small processed stub of ∼7 kDa in wild-type untagged parasites is carried into the ring stage. A small amount of full-length unprocessed proteins (370/382 kDa) found both in schizonts and in supernatants following invasion, is shown in faint outline.

The PfRh protein family plays an important role in merozoite invasion and proteolytic processing appears to be critical to their function [29]. In mature schizonts both PfRh2a and PfRh2b are processed into an 85 kDa fragment that exists as a complex with the C-terminal region of the corresponding protein. This processing event occurs post-Golgi as it is sensitive to BFA, an inhibitor of SEC7, resulting in retrograde protein transport and accumulation in the endoplasmic reticulum [24]. However, the protease cleavage of PfRh2a and b must occur before release of the merozoites from the host red blood cell as it was observed in the presence of an inhibitor of parasite egress (Figure 10). PfRh1 undergoes a similar processing event in schizonts in which the 360 kDa protein is processed to 120 kDa and 240 kDa with the larger fragment containing the erythrocyte binding region. Previously, it was not possible to show that the 120 and 240 kDa PfRh1 fragments formed a complex in schizonts, in contrast to PfRh2a and PfRh2b, and it was hypothesised that the soluble 240 kDa fragment may complex with another protein [29]. Whilst PfRh2a and PfRh2b processed complexes could be detected in both schizonts and culture supernatant there appears to be a pool of free proteins similar to the situation with PfRh1. In red cell binding experiments the 85 kDa region of PfRh2a and PfRh2b binds but there was no detectable C-terminal region suggesting either that the complex in culture supernatants may be unstable and difficult to detect or that the 85 kDa region undergoes conformational change that releases the associated C-terminal region upon binding of the receptor on the red blood cell. Our inability to detect these complexes with PfRh2a and PfRh2b antibodies other than the R2A9 polyclonal would be consistent with this possibility.

PfRh2a was processed during merozoite invasion at the transmembrane region suggesting cleavage by PfROM4 protease (Figure 10). PfROM4 has been shown to cleave the transmembrane of EBA-175, and PfRh1 and PfRh4 are also similarly cleaved resulting in the ectodomains being released into the supernatant during merozoite invasion [29], [37]. These proteins are substrates for PfROM4 when expressed in a heterologous system suggesting they are all processed by this protease during invasion [38]. It was not possible to show that PfRh2b was processed as we were unable to tag the C-terminus; however, the evidence supporting such an event for PfRh1, PfRh2a and PfRh4 suggests that it was also cleaved by PfROM4 resulting in shedding of the ectodomain from the merozoite surface [29] (Figure 10). This processing event at the end of invasion would allow disengagement of this protein family so that the merozoite can be completely encased within the parasitophorous vacuolar membrane in the early ring stage (Figure 10).

PfRh2b has been shown previously to mediate an invasion pathway into erythrocytes using a chymotrypsin-sensitive and trypsin/neuraminidase-resistant receptor Z [6]. This was defined by invasion of *P. falciparum* into enzyme-treated erythrocytes and inhibition with antibodies that specifically inhibit PfRh2b function. We have not been able to demonstrate binding of either the PfRh2a or PfRh2b C-terminal processed fragment suggesting that it could function as a scaffold on which the 85 kDa N-terminus complexes and this would allow potential signalling through the transmembrane region as has been suggested by previous work [6], [16], [44]. The 85 kDa domain of PfRh2a and b bound to erythrocytes via a trypsin-resistant and chymotrypsin/neuraminidase-sensitive receptor. The difference in neuraminidase sensitivity of the putative receptor to that observed previously may be because we are directly observing binding in comparison to previous indirect methods [6]. It is also possible that PfRh2b functions with a co-receptor and as a result the physical properties of the receptor(s) results in an average of the receptor and co-receptor attributes. The use of co-receptors for entry of viruses into host cells is very common and for example CCR5 and CXCR4 are utilised for entry of HIV-1 with different isolates varying greatly in their ability to use both [45]. It is possible that *P. falciparum* ligands are also able to mediate invasion through co-receptors although none have as yet been identified.

Previous data has shown that the PfRh2a ligand does not function in the *P. falciparum* line 3D7 despite it being expressed and capable of binding directly to erythrocytes [6]. This was shown by disruption of PfRh2b (but not PfRh2a) expression in 3D7, that resulted in abrogation of the inhibitory effect of anti-PfRh2a/b antibodies demonstrating that only PfRh2b functioned in merozoite invasion in these parasite lines. The only parasite line in which PfRh2a has been shown to function is W2mef where it appears to act to increase efficiency of invasion into neuraminidase-treated erythrocytes [30], [46]. Antibodies to the PfRh2a and b binding domains inhibit invasion of 3D7 only in the parental parasites expressing both PfRh2a and PfRh2b and also 3D7Δ2a that only expresses PfRh2b. This is in agreement with previous results demonstrating that PfRh2a in 3D7 parasites was not functional most likely due to the inactive cytoplasmic domain of this protein [6]. Once the PfRh proteins bind to the erythrocyte it has been hypothesised that they transmit a signal through the cytoplasmic tail to activate subsequent events in merozoite invasion and it is possible that this region of PfRh2a is incapable of performing this function thus inactivating this specific invasion pathway [6], [16], [44].

The receptor binding regions of PfRh1 and PfRh4 have been localised to small regions at the N-terminus of each protein [26], [27], [28]. Alignment of the binding-regions of PfRh1 and PfRh4 with other PfRh proteins has defined a semi-conserved domain and this overlaps directly with the 15 kDa region of PfRh2a and b that we have defined as the receptor binding domain [47]. This suggests that the binding of each PfRh protein is contained within the N-terminal semi-conserved region of each protein and that the rest of these large proteins act as a scaffold for display and access to this region. Previously a domain within the paralogous Py235 protein family has been identified as a ATP-binding domain [48], [49] and this plays a role in altering affinity of binding of these proteins to the receptor. Whilst it has not been shown that ATP binds to PfRh2a/b if it did in a similar way to the Py235 protein the binding site would not be located in the 85 kDa receptor binding region as it is within the C-terminal processed domain.

The PfRh proteins are located at the neck of the merozoite rhoptry [6] and must move to the apical surface before invasion to allow binding to their respective receptor on the surface of the erythrocyte [29]. Current evidence is consistent with an overlapping and cooperative function for the EBL and PfRh family suggesting they would be present in similar locations during merozoite invasion [25], [42]. Interestingly, the EBL family member EBA-175 is located in micronemes [50] and presumably it and the PfRh proteins are released at a similar time onto the apical end of the merozoite. Once at the apical end of the merozoite these proteins would be free to bind directly with specific receptors on the red blood cell and they appear to play an important role not only in the apical interaction of the merozoite but also commitment to irreversible attachment that triggers downstream events of invasion [44], [51]. Subcellular localisation of PfRh2a/b and EBA-175 during merozoite invasion of erythrocytes is consistent with an overlapping function and has suggested these proteins are located at or near the tight junction as it moves over the surface of the invading parasite. This is in agreement with previous results for the subcellular localisation of PfRh1 on invading merozoites and suggests that once these proteins bind to their receptors on the host cell they move with the tight junction during invasion and the ectodomain would be subsequently released into the culture supernatant by ROM4 cleavage. This would infer that the PfRh and EBL protein families could play an additional role(s) after apical interaction during active invasion of the host cell.

Whilst this manuscript was under review a study was published that also identified the 85 kDa processed N-terminus of PfRh2a and PfRh2b and that this fragment bound to red blood cells in agreement with our data [52]. They suggested that the C-terminal region also showed some binding; however, we have not been able to categorically show binding of this region to red blood cells in this study and our previous work [6].

The PfRh family of proteins has been shown to be a target of invasion inhibitory antibodies by the human immune system and there is evidence that this is an important component of acquired protective immunity that would act by blocking merozoite invasion [53]. Previously, it has been shown that specific antibodies to PfRh2a and b can inhibit merozoite invasion; however, these were raised to a C-terminal region of PfRh2a and b [6]. Antibodies to the receptor-binding region of PfRh2a and b also block invasion presumably by directly interfering with binding to the erythrocyte surface. It has been shown that strains of *P. falciparum* can utilise different combinations of PfRh proteins for merozoite invasion and this mechanism of phenotypic variation is mediated by differential expression and the ability of some parasites to activate specific *PfRh* genes [6], [25]. The ability of *P. falciparum* to use different invasion pathways suggests that any PfRh protein by itself would not make an effective vaccine and this is evident from the low level of growth inhibition observed for antibodies to the receptor-binding region [25], [42]. Additionally, the proteins in the PfRh family are large suggesting that specific functional regions need to be identified to which antibodies can be raised that block merozoite invasion. Indeed, recently it has been shown that immunisation of rabbits with a combination of domains from EBA-175, PfRh2a/b and PfRh4 raised antibodies that were very potent inhibiting merozoite invasion up to 90% [25], [42]. Identification of the receptor-binding site of PfRh2a and b that has been defined within a 15 kDa domain of these large proteins suggests this region would be an excellent candidate for inclusion in such a combination vaccine.

## Materials and Methods

### Ethics statement

Antibodies were raised in mice and rabbits under the guidelines of the National Health and Medical Research Committee and the PHS Policy on Humane Care and Use of Laboratory Animals. The specific details of our protocol were approved by the Royal Melbourne Hospital Animal Welfare Committee.

### Parasite cultures

*P. falciparum* parasites were maintained in human O+ erythrocytes. 3D7 is a cloned line derived from NF54 obtained from David Walliker at Edinburgh University. W2mef is a cloned line derived from the Indochina III/CDC strain and FCR3 is a cloned line. 3D7 parasites containing either a disrupted *Pfrh2a* gene (3D7Δ2a) or a disrupted *Pfrh2b* gene (3D7Δ2b) have been described previously [7]. Parasite cultures were synchronised with 5% sorbitol [54].

### Plasmids and transfection

The pHA3 plasmid enabled tagging of the *PfRh2a* gene with three HA epitopes (pHA3) and has been described previously [29]. Genomic DNA from *P. falciparum* was used as template to amplify a ∼920 bp fragment from the C-terminal end of the *PfRh2a* or *PfRh2b* genes by polymerase chain reaction (PCR) using the oligonucleotide pairs [5′AGCTagatctAAGACAAGAACAAGAACGACT]/[5′AGCTctgcagCAATTGTACTATCATTATCATTAAAAG] for *Pfrh2a* and [5′AGCTagatctGTATGATCATGTTGTTTCAGA]/[5′AGCTctgcagCAAAATATTTTTCTTCATTTTCATC] for *PfRh2b* [55] . The *Bgl II* and *Pst I* sites were used to clone the PCR fragments into the pHA3 plasmid. *P. falciparum* was transfected with 80 µg of purified plasmid DNA (Qiagen) and selection for stable transfectants by single recombination crossover was carried out as described [56].

### Cloning, fusion proteins and antibodies

To generate recombinant proteins, DNA fragments were either amplified from a codon-optimised (GeneArt, Germany) synthetic gene for N-terminal proteins (Figure S7) or from 3D7 genomic DNA by PCR and cloned into pET45. Bacterial cultures expressing the corresponding recombinant proteins were grown at 37°C to A_600_ = 0.5 then induced with IPTG at 30°C for 5 h. Cell pellets were lysed in PBS/0.1% TX100/0.5 mg/ml lysozyme/containing EDTA-free COMPLETE protease inhibitors (Roche). DNAse I was added (10 µg/ml) before sonication and centrifuged at 8000 g for 30 min at 4°C. For insoluble recombinant proteins the inclusion bodies were resuspended in PBS/0.1% TX100/0.5 mg/ml lysozyme, sonicated and washed with PBS/0.1% TX100 before solubilisation and further purification as below. Recombinant proteins that were soluble were further purified as outlined below.

The recombinant protein used to raise the antibody R1070, was generated using oligonucleotide pairs [5′AGCTggatccCAGCAACAGCGTGCTGGAT]/[5′AGCTctcgagTTAACGGTTCAGATACAGATC] and amplified from a synthetic gene (Figure S7). The insoluble recombinant protein as inclusion bodies was solubilised in 6 M guanidine/100 mM phosphate pH 8/250 mM NaCl, purified by Ni-NTA chromatography (Qiagen) and eluted with 6 M guanidine/100 mM phosphate pH 4.5/250 mM NaCl. The recombinant protein was refolded by diluting 100-fold in 2 M urea/20 mM Tris pH 8/20 mM NaCl/1 mM reduced glutathione/1 mM oxidised glutathione before further purification by ion-exchange on a HiTRAP Q (GE Healthcare) column run in 2 M urea/20 mM Tris pH 8. Elution was effected with a linear salt gradient from 20 mM to 1 M NaCl. The purified protein was dialysed against 0.5 M Urea/20 mM Tris pH 8/20 mM NaCl before injection into animals.

The rRh2_15_ recombinant protein (used to raise antibodies R1170 and 1F10) was generated using oligonucleotide pairs [5′AGCTggatccCAAAAAAAAATACGAAACCTATG]/[5′AGCTctcgagTTAATCGGTTTTTTCGATGTAGTTG] whilst the recombinant protein used to raise antibody R1088 and monoclonal antibody 8F9 was derived using oligonucleotide pairs [5′AGCTggatccCCACATAAAAAGTAAACTAGAATC]/[5′AGCTctcgagTTATGATCGAGAAAAATTTCTATC]. The soluble recombinant proteins were purified over Ni-NTA resin by washing with a buffer containing 50 mM phosphate pH 8/300 mM NaCl/10 mM imidazole and elution with 50 mM phosphate pH 8/300 mM NaCl/300 mM imidazole. Further purification was achieved by size exclusion chromatography on a Superdex 75 column (GE Healthcare).

The recombinant protein used to raise monoclonal antibody 6F12 was obtained by using the oligonucleotide pair [5′AGCTggatccCGAAAGCTATGTGATGAAC]/[5′AGCTctcgagTTAGCTGGTGTTCAGAATGG] and amplified from a synthetic gene (Figure S7). Washed inclusion bodies were solubilised in 6 M guanidine/100 mM phosphate pH8/250 mM NaCl, purified over Ni-NTA resin before elution of fusion protein with 6 M guanidine/100 mM phosphate pH 4.5/250 mM NaCl. The fusion protein was refolded by dilution 100-fold in 2 M urea/20 mM Tris pH 8/20 mM NaCl. The partially purified protein was concentrated by TCA precipitation before running in a SDS-PAGE gel and elution of the protein from the acrylamide for injection into animals.

The recombinant protein used to raise antibody R1171 was generated using the oligonucleotide pair [5′AGCTggatccCTATGTAGATGTGGACGTTTCC]/[5′AGCTctcgagTTAAATGTCCTTATTTTTTTCATCC]. Washed inclusion bodies were solubilised in 6 M guanidine/100 mM phosphate pH 8/250 mM NaCl, purified over Ni-NTA resin before elution of fusion protein with 6 M guanidine/100 mM phosphate pH 4.5/250 mM NaCl. The fusion protein was refolded by dilution 100-fold in 25 mM Tris pH7.4/20 mM NaCl, before further purification by ion-exchange on a HiTRAP Q column run in 20 mM Tris pH 7.4/20 mM NaCl. Elution was effected with a linear salt gradient from 20 mM to 1 M NaCl. The purified fusion protein was dialysed against 20 mM Tris pH 7.4/20 mM NaCl before injection into animals.

The 2b1 recombinant protein (used to raise antibody R1090 and monoclonal 4B7) was generated using oligonucleotide pairs [5′AGCTggatccCAAGATAGATGAAAGTATAACTAC]/5′AGCTctcgagTTAGTTTGAATACCTTTCATTATTG]. The soluble recombinant protein was purified over Ni-NTA resin by washing with a buffer containing 50 mM phosphate pH8/300 mM NaCl/10 mM imidazole before elution with 50 mM phosphate pH 8/300 mM NaCl/300 mM imidazole. Further purification was achieved by size exclusion chromatography on a Superdex 75 column (GE Healthcare). Final purification was achieved by ion-exchange on a HiTRAP Q column run in 20 mM Tris pH 8. Elution was effected with a linear salt gradient.

Purified fusion proteins were used to immunise rabbits and mice. Rabbit immunoglobulins were purified on Protein G-Sepharose and dialysed against PBS. Sera from mice immunised with fusion proteins were screened by ELISA against the fusion protein and by Western blot against late-stage parasite proteins. Splenic fusions from positive mice resulted in monoclonal antibodies (WEHI Monoclonal Antibody facility). The R2A9 and R2A11 antibodies have been described previously [20]. The monoclonal antibody 3A2 was raised using the GST fusion protein 2A9 previously described and used to generate R2A9 antibodies. The anti-His antibodies used were a pentaHis mouse monoclonal (Qiagen) and the anti-HA antibodies used were either the mouse monoclonal 12CA5 or the rat monoclonal 3F10 (Roche Applied Science).

### Parasite proteins, SDS-PAGE and immunoblot analysis

Culture supernatants enriched in proteins released during merozoite invasion were obtained by synchronisation of parasite cultures followed by treatment with trypsin and neuraminidase in order to prevent reinvasion of erythrocytes following schizont rupture, as described [7]. In some cases, culture supernatants were made by allowing schizont rupture of synchronised late stage cultures at high parasitaemia, without trypsin and neuraminidase treatment. Total proteins from schizont stage parasites were obtained by synchronisation and further cultured until mature schizonts were present. Parasite proteins were then obtained by saponin lysis of erythrocytes. All parasite preparations were made in the presence of COMPLETE protease inhibitors (Roche). Proteins were separated on either 3–8% Tris Acetate (for large proteins) or 4–12% Bis Tris with MES buffer (for small proteins) SDS-PAGE gels (Invitrogen). Western blotting onto nitrocellulose (0.45 µm, Schleicher and Schuell) was performed according to standard protocols and blots were processed with a chemiluminescence system (ECL, Amersham).

### *P. falciparum* radiolabelling

Mature 3D7 schizonts from highly synchronous cultures were metabolically radiolabelled with 200 uCi/ml [^35^S]methionine/cysteine as described previously [57]. To analyse the effect of brefeldin A (BFA, Sigma) on PfRh2a/b processing, BFA at 5 ug/ml or an equal volume of methanol was added to cultures for 1 h. Cultures were then spun down and resuspended in one fifth volume of methionine/cysteine-free medium, containing methanol or BFA as before and 200 µCi/ml [^35^S]methionine/cysteine for 1 h at 37°C. Labelled parasites were washed twice with PBS, uninfected erythrocytes lysed with 0.1% saponin and labelled schizonts re-washed with PBS.

To analyse the effect of E64 (trans-epoxysuccinyl-L-leucylamido-(4-guanidino)butane, Sigma) on PfRh2a/b processing, E64 at 10 µM or an equal volume of water was added to cultures under identical conditions to those described for BFA treatment.

### Pulse-chase experiments

Mature 3D7 schizonts from highly synchronous cultures were labelled for 10 min then chased as previously described [57]. Chase samples were taken at t = 0, 5, 15, 30 and 60 min and frozen directly at −70°C.

### Immunoprecipitation

Radiolabelled saponin-lysed parasites from both BFA and E64 experiments were lysed in 10 volumes TNET (1% TX100, 150 mM NaCl, 10 mM EDTA, 50 mM Tris pH7.4) containing COMPLETE (Roche) protease inhibitors. Lysates were sonicated and clarified by centrifugation. Radiolabelled pulse-chase samples were instead solubilised in five volumes of Denaturing buffer (1% SDS, 50 mM Tris pH8.0, 5 mM EDTA, COMPLETE protease inhibitors). Samples were boiled, clarified by centrifugation, then the clarified supernatants diluted 10-fold in TNET + COMPLETE inhibitors.

All immunoprecipitations of parasite proteins were carried out in a 2-step procedure by first adding antibodies, then capturing antigen:antibody complexes with ProteinG-Sepharose. Parasite proteins from schizont stages were obtained by first saponin-lysing synchronised cultures followed by schizont solubilisation with TNET. Immunoprecipitations from culture supernatants directly or from cold or radiolabelled TNET lysates were all carried out by the same 2-step procedure. Washed immunoprecipitates were separated by SDS-PAGE, blotted to nitrocellulose, then probed with antibodies of a different species to the immunoprecipitating antibodies, to avoid reactivity with immunoglobulin heavy and light chains. For experiments in Figure 5C, young Rings less than 4 h old were obtained by sorbitol synchronising a late schizont/Ring stage culture to remove schizonts. The Rings were then lysed either with 0.1% saponin or TNET, spun down, and both supernatant and pellet fractions collected. HA-containing proteins from both supernatant fractions were immunoprecipitated with anti-HA Abs. Immunoprecipitated proteins together with proteins from the saponin and TNET pellets were separated by SDS-PAGE, blotted, then probed with HRP-labelled anti-HA Abs.

### Indirect immunofluorescence and immunoelectron microscopy

Light microscopy was performed with synchronised parasites at schizont, merozoite and ring stages. Ring stages were fixed in solution with 4% paraformaldehyde and 0.0075% glutaraldehyde (ProSciTech) as described [58]. Merozoites were obtained by air-drying smears of late-stage parasites, then fixing either in 100% methanol at −20°C. Schizonts were either fixed with paraformaldehyde/glutaraldehyde or in methanol. For dual-colour fluorescence, fixed parasites in solution or on slides were blocked in 3% BSA in PBS, before both primary antibodies were added. Parasites were washed, secondary Alexa Fluor 488/594 Abs added, then mounted with VectaShield (Vector Laboratories) containing 1 µg/ml DAPI. Images were captured with a Zeiss LSM5 Live microscope with an AxioCam camera and Axiovision 4.7 software. For paraformaldehyde/glutaraldehyde-fixed parasites, single z-stacks shown were processed with AxioVision 4.7 deconvolution software. Images from methanol-fixed parasites were not deconvolved.

For immunoelectron microscopy, free or invading merozoites were fixed in 1% glutaraldehyde (ProSciTech, Australia) on ice for 30 min. Samples were pelleted in low-melt agarose before being transferred into water, dehydrated in ethanol and embedded in LR Gold Resin (ProSciTech, Australia). Following polymerization by benzoyl peroxide (SPI-Chem, USA) samples were sectioned on a Leica Ultracut R ultramicrotome (Wetzlar) and then prepared for imaging with 2% aqueous uranyl-acetate followed by 5% triple lead and observed at 120 kV on a Philips CM120 BioTWIN Transmission Electron Microscope. PfRh2 proteins were stained with the 2A9 antibody (Figure 1) while EBA-175 was stained with an antibody to the C-terminal cysteine-rich region.

### Erythrocyte binding assay

Erythrocyte binding assays using enriched culture supernatant from parasites were performed as described previously [20]. For binding assays to enzyme treated erythrocytes, erythrocytes were incubated with neuraminidase (66.7 mU/ml), high trypsin (1.0 mg/ml), Low trypsin (0.067 mg/ml) and chymotrypsin (1.0 mg/ml) for 1 hr at 37°C, then washed prior to the addition of soybean trypsin inhibitor at 0.5 mg/ml for 10 min at 37°C. Treated erythrocytes were subsequently washed three times and added to the binding assay as described above. Bound proteins eluted with 1.5 M NaCl were separated on SDS-PAGE gels, Western blotted, then probed with anti-PfRh2 or anti-EBA-175 antibodies.

### Growth inhibition assay

Growth inhibition assays were performed with modifications of a described method [59]. Briefly, twice-synchronised trophozoite stage parasites were added to either untreated target erythrocytes (at a final parasitemia of 0.5%) or to trypsin-treated erythrocytes (at a parasitemia of 1%) and haematocrit of 2% in 45 µl of culture medium in 96 well round bottom microtiter plates (Becton Dickinson, U.S.A.). 5 µl of purified antibody was added to a final concentration of 2 mg/ml. After incubation with antibodies for 1 cycle of parasite growth (∼48 h), parasites were stained with ethidium bromide (10 µg/ml final), and the parasitaemia of each well determined by flow cytometry using a FACSCalibur with a plate reader (Becton Dickinson, U.S.A.). At least two independent assays in triplicate were performed. Data was analysed using FlowJo software (Tree Star Inc). Growth was expressed as a percentage of the parasitaemia in the presence of the Protein-G purified antibodies of the prebleed serum of the same rabbit also at a final concentration of 2 mg/ml. Trypsin treatment of erythrocytes was carried out as described previously [7].

### Accession numbers

*eba-175* (MAL7P1.176); *eba-181* (JESEBL) (PFA0125c); *ebl-1* (GenBank: AAD33018.1); *eba-165* (PEBL) (PFD1155w); *eba-140* (BAEBL) (MAL13P1.60); *Pfrh1* (PFD0110w); *Pfrh2a* (PF13_0198); *Pfrh2b* (MAL13P1.176); *Pfrh3* (PFL2520w); *Pfrh4* (PFD1150c); *Pfrh5* (PFD1145c); *Pfrom4* (PFE0340c).

## Supporting Information

Figure S1 The PfRh2a and PfRh2b complexes are not held together by disulphide bonds. Supernatant proteins from the 3D7 parasite were separated by SDS-PAGE under both reducing and non-reducing conditions, then probed with antibodies to the 85 kDa fragment (R1070), a common C-terminal product (R2A9), PfRh2a (R1088) and PfRh2b (R1090). Unprocessed PfRh2b and PfRh2a are labelled as UP 2b and UP 2a respectively. The 297 kDa PfRh2b and 285 kDa PfRh2a products are also labelled. The 85 kDa fragment appears to run as a doublet under non-reduced conditions.
(EPS)

Figure S2 The 285 kDa PfRh2a and 297 kDa PfRh2b products do not bind erythrocytes. (A) Location of antibodies used in Panel B. (B) Western blots showing that the large C-terminal PfRh2a/b products do not bind erythrocytes. Both 3D7 (express both PfRh2a/b proteins) and FCR3 (do not express either PfRh2a or b) supernatant proteins were separated by SDS-PAGE and probed either with 3A2, 8F9 or 4B7 antibodies (right panels). The same supernatant proteins were also used in erythrocyte binding assays. Eluted proteins were separated by SDS-PAGE and probed with the same antibodies as above (left panels).
(EPS)

Figure S3 The PfRh proteins fall into two groups based on the alignment of conserved cysteine residues. An alignment of the first 400 amino acids of the PfRh proteins [6], [7], [18], [19], [20], [21], [22], [23] reveals a number of conserved Cys residues, shown as lines above the genes, that split the proteins into 2 groups, the PfRh2/Rh3 and the PfRh1/Rh4 groups. The PfRh2/PfRh3 group has four conserved cysteines, though the *Pfrh3* gene also has a fifth cysteine like the PfRh1/PfRh4 group. The *Pfrh3* pseudogene with a number of internal stop codons, is labelled with an asterisk since a shortened protein of only 480 amino acids is theoretically produced. The PfRh1/PfRh4 group has two closely-spaced cysteines around position 270–300. The locations of fusion proteins shown to bind erythrocytes for PfRh2a/2b, PfRh1 [7], [18], [19], [20], [22], [25], [26], [27], [28], [29] and PfRh4 are shown [7], [18], [19], [20], [22], [25], [26], [27], [28], [29].
(EPS)

Figure S4 The amino acid sequence of the rRh2_15_ fusion protein. The location of the recombinant rRh2_15_ fusion protein is shown underlined in a Figure showing an alignment of the PfRh1, PfRh4 and PfRh2a/2b proteins beginning at residue numbers 335, 328 and 393 respectively. The alignment is based on a pre-alignment of cysteine residues as shown in Figure S3. The first amino acid (Leu, position 500) of the PfRh1 binding domain and the last amino acid (Asp, position 588) of the PfRh4 binding domain is shown.
(EPS)

Figure S5 Purity of the 2b1 and rRh2_15_ fusion proteins used to bind to erythrocytes in Figure 8. Coomassie stained gels of purified fusion proteins show that 2b1 migrates at approximately 40 kDa while rRh2_15_ migrates at approximately 16 kDa on 4–12% SDS-PAGE gels.
(EPS)

Figure S6 PfRh2a, PfRh2b and PfRh1 recombinant proteins expressed and purified from *E. coli* do not bind erythrocytes. (A) Coomassie-stained gels of purified fusion proteins from PfRh2a and PfRh2b (19.1 and 45.1) and from PfRh1 (40.1). The 19.1 fusion protein corresponds to amino acids 576–718. The 45.1 fusion protein corresponds to amino acids 297–726. The 40.1 fusion protein corresponds to amino acids 50–833. (B) Purified fusion proteins detected with anti-His antibodies (left panel) and following binding and elution from erythrocytes (right panel). Also shown is binding of rRh2_15_ to red blood cells as a positive control.
(EPS)

Figure S7 Sequence of the codon-optimised PfRh2 binding domain used to PCR-amplify fragments for three N-terminal fusion proteins which led to the generation of the R1070, R1170, 1F10 and 6F12 antibodies (See Materials And Methods). All fragments were cloned in the *E. coli* expression vector, pET45.
(PDF)
