# Supplementary material for: Plasmodium falciparum Merozoite Invasion Is Inhibited by Antibodies that Target the PfRh2a and b Binding Domains
Source: PLoS Pathog. 2011 Jun 16;7(6):e1002075. doi: 10.1371/journal.ppat.1002075 (PMC3116812; doi:10.1371/journal.ppat.1002075)
Supplement: Figure S7 — Sequence of the codon-optimised PfRh2 binding domain used to PCR-amplify fragments for three N-terminal fusion proteins which led to the generation of the R1070, R1170, 1F10 and 6F12 antibodies (See Materials And Methods). All fragments were cloned in the E. coli expression vector, pET45. (PDF) [file ppat.1002075.s007.pdf]

```
GAA AGC TAT GTG ATG AAC AAC AAC CTG TAT CTG CTG CGT GTG AAC GAA GTG AAA AGC ACC CCG ATT GAT CTG TAT CTG AAC CGT GCG AAA GAA CTG CTG G  < 100
E   S   Y   V   M   N   N   N   L   Y   L   L   R   V   N   E   V   K   S   T   P   I   D   L   Y   L   N   R   A   K   E   L   L   E
CTT TCG ATA CAC TAC TTG TTG TTG GAC ATA GAC GAC GCA CAC TTG CTT CAC TTT TCG TGG GGC TAA CTA GAC ATA GAC TTG GCA CGC TTT CTT GAC GAC C
                10          20          30          40          50          60          70          80          90

AA AGC AGC AGC AAA CTG GTG AAT CCG ATC AAA ATG AAA CTG GGC GAT AAC AAA AAC ATG TAC AGC ATC GGC TAT ATC CAC GAT GAA ATC AAA GAT ATC AT  < 200
   S   S   S   K   L   V   N   P   I   K   M   K   L   G   D   N   K   N   M   Y   S   I   G   Y   I   H   D   E   I   K   D   I   I
TT TCG TCG TCG TTT GAC CAC TTA GGC TAG TTT TAC TTT GAC CCG CTA TTG TTT TTG TAC ATG TCG TAG CCG ATA TAG GTG CTA CTT TAG TTT CTA TAG TA
               110         120         130         140         150         160         170         180         190

C AAA CGC TAT AAC TTC CAC CTG AAA CAC ATC GAA AAA GGC AAA GAA TAT ATT AAA CGC ATC ACC CAG GCG AAC AAC ATT GCG GAT AAA ATG AAA AAA GAT  < 300
  K   R   Y   N   F   H   L   K   H   I   E   K   G   K   E   Y   I   K   R   I   T   Q   A   N   N   I   A   D   K   M   K   K   D
G TTT GCG ATA TTG AAG GTG GAC TTT GTG TAG CTT TTT CCG TTT CTT ATA TAA TTT GCG TAG TGG GTC CGC TTG TTG TAA CGC CTA TTT TAC TTT TTT CTA
               210         220         230         240         250         260         270         280         290

GAA CTG ATC AAA AAA ATC TTC GAA AGC AGC AAA CAT TTT GCG AGC TTC AAA TAT AGC AAC GAA ATG ATC AGC AAA CTG GAT AGC CTG TTC ATT AAA AAT G  < 400
E   L   I   K   K   I   F   E   S   S   K   H   F   A   S   F   K   Y   S   N   E   M   I   S   K   L   D   S   L   F   I   K   N   E
CTT GAC TAG TTT TTT TAG AAG CTT TCG TCG TTT GTA AAA CGC TCG AAG TTT ATA TCG TTG CTT TAC TAG TCG TTT GAC CTA TCG GAC AAG TAA TTT TTA C
               310         320         330         340         350         360         370         380         390

AA GAA ATC CTG AAC AAC CTG TTC AAC AAC ATC TTT AAC ATC TTC AAA AAA AAA TAC GAA ACC TAT GTG GAT ATG AAA ACC ATC GAA AGC AAA TAT ACC AC  < 500
   E   I   L   N   N   L   F   N   N   I   F   N   I   F   K   K   K   Y   E   T   Y   V   D   M   K   T   I   E   S   K   Y   T   T
TT CTT TAG GAC TTG TTG GAC AAG TTG TTG TAG AAA TTG TAG AAG TTT TTT TTT ATG CTT TGG ATA CAC CTA TAC TTT TGG TAG CTT TCG TTT ATA TGG TG
               410         420         430         440         450         460         470         480         490

C GTG ATG ACC CTG AGC GAA CAT CTG CTG GAA TAT GCC ATG GAT GTG CTG AAA GCG AAT CCG CAG AAA CCG ATT GAT CCG AAA GCG AAC CTG GAT AGC GAA  < 600
  V   M   T   L   S   E   H   L   L   E   Y   A   M   D   V   L   K   A   N   P   Q   K   P   I   D   P   K   A   N   L   D   S   E
G CAC TAC TGG GAC TCG CTT GTA GAC GAC CTT ATA CGG TAC CTA CAC GAC TTT CGC TTA GGC GTC TTT GGC TAA CTA GGC TTT CGC TTG GAC CTA TCG CTT
               510         520         530         540         550         560         570         580         590

GTG GTG AAA CTG CAG ATC AAA ATT AAC GAA AAA AGC AAC GAA CTG GAT AAC GCG ATT AGC CAG GTG AAA ACC CTG ATC ATC ATC ATG AAA AGC TTC TAT G  < 700
V   V   K   L   Q   I   K   I   N   E   K   S   N   E   L   D   N   A   I   S   Q   V   K   T   L   I   I   I   M   K   S   F   Y   D
CAC CAC TTT GAC GTC TAG TTT TAA TTG CTT TTT TCG TTG CTT GAC CTA TTG CGC TAA TCG GTC CAC TTT TGG GAC TAG TAG TAG TAC TTT TCG AAG ATA C
               610         620         630         640         650         660         670         680         690

AT ATC ATC ATC AGC GAA AAA GCG AGC ATG GAT GAA ATG GAA AAA AAA GAA CTG AGC CTG AAC AAC TAC ATC GAA AAA ACC GAT TAT ATC CTG CAG ACC TA  < 800
   I   I   I   S   E   K   A   S   M   D   E   M   E   K   K   E   L   S   L   N   N   Y   I   E   K   T   D   Y   I   L   Q   T   Y
TA TAG TAG TAG TCG CTT TTT CGC TCG TAC CTA CTT TAC CTT TTT TTT CTT GAC TCG GAC TTG TTG ATG TAG CTT TTT TGG CTA ATA TAG GAC GTC TGG AT
               710         720         730         740         750         760         770         780         790

T AAC ATC TTT AAA AGC AAA AGC AAC ATC ATC AAC AAC AAC AGC AAA AAC ATC AGC AGC AAA TAC ATT ACC ATC GAA GGC CTG AAA AAC GAT ATC GAT GAA  < 900
  N   I   F   K   S   K   S   N   I   I   N   N   N   S   K   N   I   S   S   K   Y   I   T   I   E   G   L   K   N   D   I   D   E
A TTG TAG AAA TTT TCG TTT TCG TTG TAG TAG TTG TTG TTG TCG TTT TTG TAG TCG TCG TTT ATG TAA TGG TAG CTT CCG GAC TTT TTG CTA TAG CTA CTT
               810         820         830         840         850         860         870         880         890

CTG AAC AGC CTG ATC AGC TAT TTT AAA GAT AGC CAG GAA ACC CTG ATT AAA GAC GAT GAG CTG AAA AAA AAT ATG AAA ACC GAT TAC CTG AAC AAC GTG A  < 1000
L   N   S   L   I   S   Y   F   K   D   S   Q   E   T   L   I   K   D   D   E   L   K   K   N   M   K   T   D   Y   L   N   N   V   K
GAC TTG TCG GAC TAG TCG ATA AAA TTT CTA TCG GTC CTT TGG GAC TAA TTT CTG CTA CTC GAC TTT TTT TTA TAC TTT TGG CTA ATG GAC TTG TTG CAC T
               910         920         930         940         950         960         970         980         990

AA TAT ATC GAA GAA AAC GTG ACC CAT ATC AAC GAA ATC ATC CTG CTG AAA GAT AGC ATT ACC CAG CGT ATT GCG GAT ATC GAC GAG CTG AAT TCT CTG AA  < 1100
   Y   I   E   E   N   V   T   H   I   N   E   I   I   L   L   K   D   S   I   T   Q   R   I   A   D   I   D   E   L   N   S   L   N
TT ATA TAG CTT CTT TTG CAC TGG GTA TAG TTG CTT TAG TAG GAC GAC TTT CTA TCG TAA TGG GTC GCA TAA CGC CTA TAG CTG CTC GAC TTA AGA GAC TT
              1010        1020        1030        1040        1050        1060        1070        1080        1090

C CTG ATC AAC ATC AAC GAT TTC ATC AAC GAA AAA AAC ATT AGC CAG GAA AAA GTT AGC TAT AAC CTG AAT AAA CTG TAT AAA GGC AGC TTC GAA GAA CTG  < 1200
  L   I   N   I   N   D   F   I   N   E   K   N   I   S   Q   E   K   V   S   Y   N   L   N   K   L   Y   K   G   S   F   E   E   L
G GAC TAG TTG TAG TTG CTA AAG TAG TTG CTT TTT TTG TAA TCG GTC CTT TTT CAA TCG ATA TTG GAC TTA TTT GAC ATA TTT CCG TCG AAG CTT CTT GAC
              1110        1120        1130        1140        1150        1160        1170        1180        1190

GAA AGC GAA CTG AGC CAT TTT CTG GAT ACC AAA TAC CTG TTT CAC GAA AAA AAA AGC GTG AAC GAA CTG CAG ACC ATT CTG AAC ACC AGC  < 1290
E   S   E   L   S   H   F   L   D   T   K   Y   L   F   H   E   K   K   S   V   N   E   L   Q   T   I   L   N   T   S
CTT TCG CTT GAC TCG GTA AAA GAC CTA TGG TTT ATG GAC AAA GTG CTT TTT TTT TCG CAC TTG CTT GAC GTC TGG TAA GAC TTG TGG TCG
              1210        1220        1230        1240        1250        1260        1270        1280
```
